# Do MCI patients with vitamin B12 deficiency have distinctive cognitive deficits?

**DOI:** 10.1186/1756-0500-6-357

**Published:** 2013-09-08

**Authors:** Dina Silva, Ulrike Albers, Isabel Santana, Margarida Vicente, Isabel Pavão Martins, Ana Verdelho, Manuela Guerreiro, Alexandre de-Mendonça

**Affiliations:** 1 Dementia Clinics, Faculty of Medicine, University of Lisbon, Lisbon, Portugal; 2 Department of Health and Human Performance, Faculty of Physical Activity and Sport Sciences-INEF, Universidad Politécnica de Madrid, Madrid, Spain; 3 Department of Neurology, Coimbra University Hospital, Coimbra, Portugal; 4 Laboratory of Language Research, Faculty of Medicine, University of Lisbon, and Hospital de Santa Maria, Lisbon, Portugal; 5 Department of Neurology, Santa Maria Hospital, Lisbon, Portugal; 6 Institute of Molecular Medicine and Faculty of Medicine, University of Lisbon, Lisbon, Portugal; 7 Instituto de Farmacologia e Neurociências, Edifício Egas Moniz, Faculdade de Medicina de Lisboa, Avenida Prof. Egas Moniz, 1649-028 Lisboa, Portugal

**Keywords:** Mild cognitive impairment, Cognitive decline, Vitamin B12 deficiency, Memory impairment, Neuropsychological tests

## Abstract

**Background:**

Vitamin B12 deficiency is common in older people, and may be responsible for reversible dementia. Low serum vitamin B12 levels were also observed in patients with Mild Cognitive Impairment (MCI). It is not known whether patients with vitamin B12 deficiency have a distinctive profile of cognitive impairment different from the episodic memory deficit usually observed in MCI.

**Results:**

From a cohort of 310 patients with MCI followed in a memory clinic in Lisbon, only 10 cases with vitamin B12 deficiency were found. From collaboration with other neurologists, 5 further patients with vitamin B12 deficiency were added. These cases were compared to MCI patients with normal vitamin B12 levels in a ratio 1:3. The duration of subjective cognitive symptoms was significantly shorter in MCI patients with B12 deficiency (1.2±1.0 years) as compared to MCI patients with normal vitamin B12 levels (3.4±3.0 years, *p*<0.001, Student’ *t* test). There were no statistically significant differences in the neuropsychological tests between MCI patients with and without vitamin B12 deficiency. Vitamin B12 was started in MCI patients with vitamin B12 deficiency, with no noticeable clinical improvement.

**Conclusion:**

MCI patients with low levels of vitamin B12 had no particular profile of cognitive impairment, however vitamin B12 deficiency might have precipitated the onset of symptoms. The effect of vitamin B12 supplementation in patients with MCI and low vitamin B12 levels should be clarified by future prospective studies.

## Background

Vitamin B12 deficiency is common in the elderly, affecting as much as 10% to 15% of people over the age of 60 as a consequence of inadequate intake or malabsortion [1]. Vitamin B12 deficiency causes a classical neurological and hematological syndrome [2,3], and has long been associated with cognitive and psychiatric disturbances [4]. In a review of 32 studies, vitamin B12 deficiency was responsible for about 1% of the reversible dementias [5].

More recently, vitamin B12 deficiency was also implicated in milder forms of cognitive decline. In a consecutive series of patients observed in a memory clinic, 3.3% of patients with Mild Cognitive Impairment (MCI) had low serum values of vitamin B12 [6]. MCI is usually considered a transitional state between normal cognitive aging and dementia [7]. Although patients with MCI may have involvement of different cognitive domains, episodic memory is most consistently disturbed [8]. It could be that patients with vitamin B12 deficiency had a distinctive profile of cognitive impairment, since cognitive domains other than memory were reported to be affected by the deficiency of vitamin B12. In a previous study, non-demented cognitively impaired elderly patients with vitamin B12 deficiency had lower verbal fluency scores as compared with those with normal values of vitamin B12 [9]. In a sample of non-demented elderly subjects from a community based study, both normal and cognitively impaired, the values of methylmalonic acid, which inversely reflect the levels of vitamin B12, were associated with worse performances in language and praxis tests [10]. In another study performed in non-demented elderly subjects, low concentrations of vitamin B12 were associated with poorer performance in a spatial copying test [11]. Also in a sample of non-demented subjects older than 75, low levels of vitamin B12 were associated with decreased performance in a modified block design test, which evaluates abstraction and visuospatial abilities [12]. Thus, different cognitive domains were reported to be affected in non-demented elderly subjects with vitamin B12 deficiency.

In the present study, we hypothesized that patients with cognitive impairment and vitamin B12 deficiency may have a particular profile of cognitive deficits that could be different from the usual pattern of episodic memory impairment observed in MCI patients. To test this hypothesis, we compared the neuropsychological performances of MCI patients with vitamin B12 deficiency to neuropsychological performances of matched MCI patients with normal serum vitamin B12 levels. Recognition of a particular neuropsychological profile could indicate preferential requirements or a particular role for vitamin B12 in specific brain areas, and possibly facilitate the detection of vitamin B12 deficiency in patients with MCI.

## Methods

The database of a Memory Clinic in Lisbon was systematically searched for patients with the diagnosis of MCI for the period 2002–2010. Since a limited number of patients with vitamin B12 deficiency was found, and for the sake of an appropriate sample size, other neurologists (IS, IPM, AV) were requested and agreed to provide cases with cognitive deficits and vitamin B12 deficiency. All patients were observed in a dementia outpatient clinic setting due to the presence of cognitive complaints, and underwent a standard and comprehensive neuropsychological assessment by the same neuropsychologists (MG, DS, MV).

The study was approved by the *Comissão de Ética para a Saúde do Hospital de Santa Maria*.

### Inclusion criteria

Mild Cognitive Impairment was diagnosed according to the criteria by the MCI Working Group of the European Consortium on Alzheimer’s disease [13]:

1. Cognitive complaints coming from the patients or their families;

2. Reporting of a decline in cognitive functioning relative to previous abilities during the past year by the patient or informant;

3. Objective impairment in memory or another cognitive domain;

4. Absence of major repercussions in daily life (the patient may report difficulties concerning complex day-to-day activities).

### Vitamin B12 deficiency

Patients with MCI were considered to have vitamin B12 deficiency when vitamin B12 serum levels were less than 200 pg/mL, and to have normal vitamin B12 if the serum levels were equal or higher than 200 pg/mL [14,15].

### Selection of cases and controls

Clinical files of all MCI patients were reviewed at the Memory Clinic in Lisbon (2002–2010). The cases with vitamin B12 deficiency were selected. Cases with vitamin B12 deficiency and neuropsychological assessment referred from the other participating neurologists were also included. For each patient presenting vitamin B12 deficiency, 3 MCI subjects with normal levels of vitamin B12, matched for gender, education and age (± 2 years), were consecutively selected for controls from the clinical files of the Memory Clinic. The selection ratio 1:3 enhanced the power to detect differences in the neuropsychological tests between the two groups, given a limited number of patients with vitamin B12 deficiency.

### Exclusion criteria

1. Diagnosis of neurological or psychiatric disorders that might justify the cognitive deficits associated to the MCI condition;

2. History of alcohol abuse or recurrent substance abuse or dependence;

3. Systemic illness with cerebral involvement, untreated endocrine disorders, metabolic deficits other than vitamin B12 (for instance folate deficiency);

4. Presence of dementia according to DSM-IV-TR [16];

5. Vitamin B12 levels not obtained or not recorded.

### Neuropsychological assessment

The detailed neuropsychological assessment was carried out by the same team of trained neuropsychologists with the *Battery of Lisbon for the Assessment of Dementia* (BLAD) [17], a comprehensive neuropsychological battery evaluating multiple cognitive domains and validated for the Portuguese population. This battery includes tests for the following cognitive domains: attention and executive functions (*Cancellation Task, Digit Span backward* and *Clock Draw*); initiative (*Verbal Semantic Fluency*, *Motor Initiative* and *Graphomotor Initiative*); conceptual thinking (*Raven Progressive Matrices* and *Interpretation of Proverbs*); calculation (*Basic Written Calculation*); language (a modified version of the *Token Test*); personal, spatial and temporal orientation (*Orientation Questionnaire*); immediate memory (*Digit Span forward*); episodic memory and associative learning (*Verbal Paired-associate Learning*, *Logical Memory* and *Word Recall*). A Forgetting Index was also calculated based on the correct information evoked between the immediate and the delayed condition of the Logical Memory Test (Forgetting Index= [(LM delayed recall – LM immediate) / LM immediate)] × 100) [18].

Depressive symptoms were evaluated with the *Geriatric Depression Scale* (GDS) [19,20], a self-report assessment used specifically to identify depression in the elderly. For this study a short-form (15 items) of the self-report instrument was used [21].

### Statistical analyses

The sample size was calculated to test the hypothesis that patients with vitamin B12 deficiency have less deficits in episodic memory as compared to typical patients with MCI. In a previous paper, we found that patients with MCI had a score in Logical Memory (immediate recall) of −1.74±0.89 (z score) [22]. Assuming that patients with vitamin B12 deficiency would score higher than −1 SD, and for a significance level of 0.05, a power of 0.80 and a ratio B12 deficiency:control of 1:3, a total sample size of 60 (15:45) would be needed [23].

The presence of statistically significant differences between groups in demographic and clinical data was analysed using Student’s *t* test for numerical data and the Fisher’s exact test for categorical data. Since several neuropsychological tests scores did not follow the normal distribution, the Mann–Whitney test was used to compare performances in neuropsychological tests of MCI patients with and without B12 deficiency. No correction for multiple comparisons was done. Statistical significance was set at *p* values < 0.05. All statistical analyses were performed using SPSS for Windows (PASW Statistics 18; SPSS Inc, Chicago, USA).

## Results

The clinical files of 310 patients with the diagnosis of MCI evaluated in a Memory Clinic in Lisbon were reviewed. Only 10 MCI patients with B12 deficiency were found. It was possible to add 5 further cases of MCI patients with vitamin B12 deficiency from the other participant neurologists to achieve the sample size needed for the power and significance established. None of these cases had anemia or neurological symptoms other than cognitive.

Demographic data are shown in Table 1, no statistically significant differences being found between MCI patients with B12 deficiency and MCI patients with normal vitamin B12 levels, as expected from the matching procedure. The duration of cognitive symptoms was significantly shorter in MCI patients with B12 deficiency (1.2±1.0 years) as compared to MCI patients with normal vitamin B12 levels (3.4±3.0 years, *p*<0.001, Student’ *t* test, Table 1). There were no significant differences concerning the presence of depressive symptoms and the prescription of drugs with possible interference on cognition, namely anticholinergic effects (Table 1). As expected, the levels of serum vitamin B12 were significantly different between the two groups (*p*<0.001, Student’ *t* test, Table 1).

**Table 1:** Demographic and clinical data of patients with Mild Cognitive Impairment (MCI) and B12 deficiency compared with patients with MCI and normal vitamin B12 levels

	**MCI vitamin B12 deficiency (n=15)**	**MCI normal vitamin B12 (n=45)**	***P *- value**
**Age,** years [mean ± SD (range)]	74.9±7.6	74.9±6.9	1.00^‡^
**Sex,** female/male (n)	7/8	22/23	1.00^**†**^
**Formal education,** years (mean ± SD)	6.7±4.6	7.4±4.8	0.66^‡^
**Vitamin B12 level,** pg/mL (mean ± SD)	148.4±42.7	496.9±218.9	<0.001^‡^*
**Duration of cognitive complaints** (mean ± SD)	1.2±1.0	3.4±3.0	<0.001^‡^*
**Depressive symptoms, geriatric depression scale** (mean ± SD)	3.6±1.5	4.1±2.9	0.59^‡^
**Drugs with possible effect on cognition** (n)	0	5	0.32^**†**^
**Response to vitamin B12 therapy,** Yes/No/Unknown (n)	0/14/1	NA	

^‡^Student’s t-test, ^†^Fisher’s exact test; *Statistically significant (*p*< 0.05), *NA* not applicable

The results of the neuropsychological tests of MCI patients with vitamin B12 deficiency were compared with the results of matched MCI patients with normal vitamin B12 levels, and no statistically significant differences were found, even without adjusting the significance levels for multiple comparisons (Table 2). The most pronounced deficits were found in memory tests and orientation, for both groups (Table 2).

**Table 2:** Comparison of the neuropsychological performances of MCI patients with vitamin B12 deficiency with the performances of MCI patients with normal vitamin B12 levels

**Cognitive domain**	**MCI vitamin B12 deficiency (n=15)**	**MCI normal vitamin B12 (n=45)**	***p *– value**^**‡**^
**Neuropsychological tests**	**# mean ± SD**	**# mean ± SD**	
**Attention and executive functions**			
Cancellation task	−0.004±0.98	0.07±0.97	0.90
Digit span backward	0.78±1.07	0.24±0.72	0.13
Clock draw	0.80±0.71	0.59±0.89	0.84
**Initiative**			
Verbal semantic fluency	−0.22±1.40	−0.55±1.40	0.30
Motor initiative	0.59±0.57	0.12±1.40	0.51
Graphomotor initiative	0.24±0.50	0.10±0.71	0.58
**Conceptual thinking**			
Raven progressive matrices	−0.08±0.90	0.10±0.97	0.62
Interpretation of proverbs	1.03±0.68	0.36±1.00	0.09
**Calculation**			
Basic written calculation	−0.18±0.78	−0.16±1.08	0.62
**Language**			
Token test	0.24±1.05	−0.12±1.51	0.74
**Orientation**			
Personal, spatial and temporal	−1.99±2.76	−1.67±2.29	0.88
**Immediate memory**			
Digit span forward	0.87±1.52	0.64±1.34	0.71
**Episodic memory and associative learning**			
Verbal paired-associate learning	−0.70±1.23	−1.42±0.65	0.11
Logical Memory (LM; immediate recall)	−1.37±1.43	−1.64±0.93	0.46
Word recall	−0.89±1.11	−1.30±1.24	0.45
Forgetting index^**†**^	−1.26±0.97	−0.67±2.34	0.55

**#***z* scores are shown; data are standardized according to the age and education norms from the Portuguese population.

^**†**^ Forgetting Index = [(LM delayed recall – LM immediate) / LM immediate)]*100.

^**‡**^ Mann–Whitney test.

Vitamin B12 therapy was started in all MCI patients with vitamin B12 deficiency (1 mg orally every day or 1 mg by intramuscular administration every month) and the normalization of serum levels was obtained. However, the clinical follow-up (6 months to 3 year after starting supplementation; one patient out of the 15 was lost to follow-up) did not show clinical benefits from vitamin B12 therapy.

## Discussion

Patients with MCI and vitamin B12 deficiency had no particular profile of cognitive impairment that would be distinct from that usually observed in MCI patients with normal vitamin B12 levels. Episodic memory and orientation were predominantely affected in MCI patients with vitamin B12 deficiency, which are the cognitive domains typically altered in MCI patients [7,8]. On the other hand, cognitive domains previously reported to be disturbed by vitamin B12 deficiency, namely verbal fluency [9], language and praxis [10], and abstraction and visuospatial abilities [11,12] were not more impaired in MCI patients with vitamin B12 deficiency as compared to MCI patients with normal vitamin B12 levels.

Several cross-sectional and longitudinal studies in both healthy and cognitively impaired older subjects reported inverse associations between vitamin B12 levels and the degree of cognitive impairment [24-27], whereas other studies found no association [28-31]. Moreover, a recent review found that vitamin B12 deficiency is associated with cognitive impairment, but supplementation did not improve cognitive function in patients with previous deficits [32].

The benefit of vitamin B12 therapy regarding cognition is presently not clear. There are reports of patients with cognitive deficits who improved, namely in language and frontal lobe functions, after vitamin B12 supplementation [9]. However, the hypothesis that supplementation of vitamin B12, together with other vitamins, could have beneficial cognitive effects in subjects with normal or impaired cognition was not demonstrated neither in a randomized controlled trial [33] nor in a recent systematic review and meta-analysis [34].

Some authors have raised the question whether the presence of low levels of vitamin B12 in age-associated cognitive impairment might be an epiphenomenon, or a consequence of disease progression, rather than the cause of cognitive decline [26,35-37]. In the present study, the finding of similar neuropsychological profiles for MCI patients with low vitamin B12 levels, as compared to those with normal levels, would suggest that low vitamin B12 was just an incidental finding. This interpretation was also supported by the observation that the patients with vitamin B12 deficiency prescribed high doses of vitamin B12 (either oral or intramuscular see [38,39]), did not clinically improve, even though the duration of cognitive symptoms before supplementation was within the time window (about 1 year) proposed for a successful supplementation therapy [40,41]. On the other hand, the finding of a shorter duration of clinical symptoms in MCI patients with vitamin B12 deficiency may suggest that this metabolic disturbance could somehow have precipitated the onset, or even accelerated the progression, of cognitive symptoms in patients diagnosed with MCI.

Limitations of the study should be recognized. It had a retrospective design, which might have introduced a selection bias, and would not allow the recognition of a possible distinct clinical evolution for the MCI patients with vitamin B12 deficiency. Regarding the effect of vitamin B12 supplementation, one patient had no follow-up information, and others might not have been followed long enough to detect a sizeable response to therapy. Also, a formal neuropsychological re-assessment was not performed which might detect eventual subtle improvements in some cognitive tests after vitamin B12 supplementation.

Another limitation is that methylmalonic acid, which may reflect more closely the physiological role of vitamin B12, was not quantified. Since conversion of methylmalonyl-coenzyme A to succinyl-coenzyme A depends upon vitamin B12, increased levels of methylmalonic acid are considered a sensitive and specific marker for functional vitamin B12 deficiency [10].

It is interesting to note that, in spite of vitamin B12 being tested as part of the standard laboratory diagnostic workup for patients with cognitive complaints at the Memory Clinic in Lisbon, only 3.2% of all MCI patients observed in this institution during the period of 2002 to 2012 had decreased serum values of vitamin B12, confirming a low frequency of vitamin B12 deficiency in MCI patients [6]. Nowadays, in clinical practice, the prevalence of reversible dementias is declining [42] and the identified metabolic abnormalities are often not the main cause responsible for the cognitive symptoms when compared with the neurodegenerative pathology [43]. Nevertheless, detection of a metabolic disturbance, like vitamin B12 deficiency, is still advisable, since it might contribute to cognitive deterioration in individual cases and is usually amenable to correction. Moreover, vitamin B12 deficiency appears to precipitate the onset of symptoms in patients with MCI. The effect of vitamin B12 supplementation in patients with MCI and low vitamin B12 levels should be clarified by future prospective studies.

## Conclusions

The profile of cognitive impairment in MCI patients with and without vitamin B12 deficiency was similar, suggesting that the finding of low vitamin B12 serum levels in some cases of MCI could be just coincidental. Interestingly, the MCI patients with low vitamin B12 serum levels had a shorter duration of clinical symptoms than MCI patients with normal levels of vitamin B12, raising the hypothesis that this metabolic disturbance could have somehow precipitated the onset of cognitive symptoms in those patients. Future prospective studies are needed to clarify the effect of vitamin B12 supplementation on the clinical progression of patients with MCI and low vitamin B12 levels.

### Ethics statement

The study was conducted in accordance with the Declaration of Helsinki, and the local ethics committee, *Comissão de Ética para a Saúde do Hospital de Santa Maria*, approved the study.

## Competing interests

The authors declare no competing interests.

## Authors’ contributions

DS participated in the coordination of the study, selected the patients from the clinical database, conducted part of the neuropsychological assessment of MCI controls, performed statistical analysis and contributed to draft the manuscript. UA conceived the study and participated in its design. IS, IPM and AV selected MCI patients with vitamin B12 deficiency enrolled in the study, implemented and assessed the response to vitamin B12 replacement. MV conducted the neuropsychological assessments of MCI patients from Coimbra University Hospital. MG participated in the design of the study and coordinated the neuropsychological evaluations of MCI patients in Lisbon. AM conceived the study, participated in its design and coordination, selected the patients with vitamin B12 deficiency enrolled in the study and contributed to draft the manuscript. All authors read and approved the final manuscript.
